# Experimental demonstration of broadband impedance matching using coupled electromagnetic resonators

**DOI:** 10.1038/s41598-020-64439-w

**Published:** 2020-05-04

**Authors:** Xiaolong Lv, Chuanfei Li, Yaohua Que, Guofeng Li, Xiaojuan Hou, Ying Li, Linfeng Li, Yibo Sun, Yunsheng Guo

**Affiliations:** School of Science, Inner Mongolia University of Science and Technology, Baotou, 014010 China

**Keywords:** Materials science, Optics and photonics

## Abstract

Impedance matching is an important factor for the electromagnetic resonators used to construct metasurfaces with perfect absorption and transmission properties. However, these resonators usually exhibit narrowband characteristics, thus greatly restricting their potential for application to metasurfaces to obtain excellent absorption and transmission performances. Therefore, realization of impedance matching over a wider range is of major importance. In this work, we demonstrate broadband impedance matching both theoretically and experimentally through use of coupled inductor-capacitor (LC) resonant coils, which are typical electromagnetic resonators. By adding a third resonant coil into the conventional system composed of two completely mismatched resonant coils, the new system realizes broadband impedance matching when the reflected impedances of the first two coils with respect to the third resonant coil are equal. The results in this work can provide useful guidance for realization of metasurfaces with broadband perfect absorption and transmission constructed using any type of electromagnetic resonator.

## Introduction

Reflection, transmission, and absorption are very important aspects of research in the fields of electromagnetic and optical engineering. The design and construction of excellent quality materials or components that can reflect, transmit, or absorb external electromagnetic radiation completely is an aim that is constantly pursued by researchers^1–5^. Fortunately, the main design and construction issues were basically solved when metasurfaces were devised^6–8^. Metasurfaces are artificially fabricated 2D periodic structures composed of subwavelength electromagnetic resonators that can control the amplitude, phase, and polarization of incident electromagnetic waves arbitrarily and exhibit powerful and unusual electromagnetic properties that do not exist in natural materials^9–11^. Therefore, the realization of perfect reflection, transmission, and absorption properties using metasurfaces is a very promising option. In fact, metasurface-based perfect absorbers, reflectors and transmitters have been reported extensively in the microwave^12–18^, terahertz^19–25^, infrared^26–31^, and visible light^32–34^ bands over the past decade. All these perfect performances are derived from the resonance responses of the electromagnetic resonators used in their construction, which are the source of the unusual properties of metasurfaces. However, because of the nature of these resonance responses, the resulting metasurfaces usually show very narrow operating bands^35^. In addition, when compared with perfect reflectors, the options for realization of perfect absorbers and transmitters are more restricted because their impedances, which are determined by the resonators, should be matched with that of free space^36^. Therefore, adjustment of the impedance of the resonators, and particularly impedance matching within a broadband range, has greater significance.

To date, our group has proposed different approaches to achieve broadband impedance matching of electromagnetic resonators. For example, using localized E-field coupling of two split-ring resonators^37^ and the coupled Mie resonance of two high-permittivity low-loss ceramic particles^38^, the impedances at the two ends of a rectangular waveguide blocked by a subwavelength metal aperture are very well matched over a wide range and total broadband transmission was thus obtained. However, this approach was only applicable in cases where subwavelength metal apertures were placed within the waveguides. Recently, broadband impedance matching and perfect absorption based on coupled semiconductor resonators has been studied in detail^39^, but the corresponding experimental studies have not been carried out to date. In this work, we demonstrate both experimentally and theoretically the feasibility of broadband impedance matching using coupled inductor-capacitor (LC) resonators. When a third LC resonator is introduced into the system composed of the two mismatched LC resonators, the original two resonators can then be matched very well over a suitably wide frequency range. The LC resonator is the most basic electromagnetic resonator available and can be applied to construction of metasurfaces and to other fields, e.g., wireless power transfer^40,41^; therefore, the conclusions drawn here can be used directly to guide broadband impedance matching of other electromagnetic resonators over the entire electromagnetic spectrum.

## Results

### Matching of two-coil system

**Figure 1:**
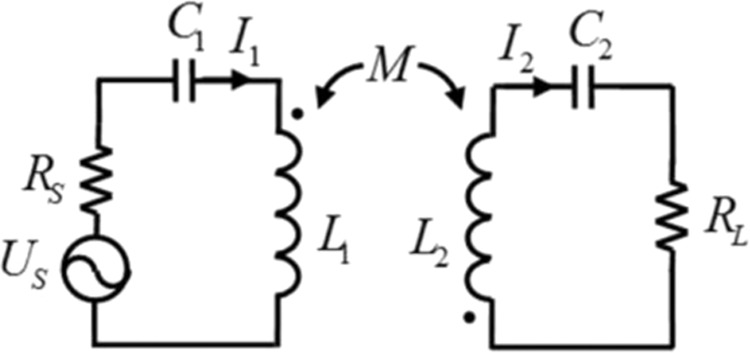
Equivalent circuit of the two coupled inductor-capacitor (LC) resonant coils.

Resonance is a very common phenomenon that occurs in nature. Spring masses and LC circuits are generally adopted as models to study the physical parameters related to resonance. Here, the LC circuit model is used to verify the feasibility of broadband impedance matching of coupled electromagnetic resonators. Figure 1 shows the equivalent circuit of the two LC coils when coupled via their magnetic fields. The left side is the transmitting coil, and the right side shows the receiving coil. The inductances of the transmitting and receiving coils and the mutual inductance between these coils are L_1_, L_2_ and M, respectively. The capacitances C_1_ and C_2_ are connected with the transmitting and receiving coils, respectively, to achieve resonance. A power source with electromotive force U_S_ and internal resistance R_S_ is connected to the transmitting coil. The load resistance R_L_ is connected to the receiving coil. The currents that flow through the transmitting and receiving coils are I_1_ and I_2_, respectively. According to Kirchhoff’s law, the relationship between I_1_ and I_2_ can be given as

1 $$[\begin{array}{c}{U}_{S}\\ 0\end{array}]=[\begin{array}{cc}{Z}_{1} & j\omega M\\ j\omega M & {Z}_{2}\end{array}][\begin{array}{c}{I}_{1}\\ {I}_{2}\end{array}]$$

In the matrix in Equation (1), *Z*_1_ and *Z*_2_ represent the impedances of the closed circuits formed by the transmitting and receiving coils, respectively, and are given by:

2 $${Z}_{1}={R}_{S}+j(\omega {L}_{1}-1/\omega {C}_{1})$$

3 $${Z}_{2}={R}_{L}+j(\omega {L}_{2}-1/\omega {C}_{2})$$

Note that the radiation and the coil resistance are ignored in Fig. 1 and in Eqs. (2) and (3). The currents that were derived from Eq. (1) are

4 $${I}_{1}=\frac{{U}_{S}}{{Z}_{1}+{(\omega M)}^{2}/{Z}_{2}}$$

5 $${I}_{2}=\frac{-j\omega M{I}_{1}^{{\prime} }}{{Z}_{2}+{(\omega M)}^{2}/{Z}_{1}}$$

where *I*′_1_ = *U*_S_*/Z*_1_ is the transmitting coil current in the absence of the receiving coil. From the expression given in Eq. (4), we know that the presence of the receiving coil causes a reflected impedance, (*ωM*)^2^/*Z*_2_, for the transmitting coil. Similarly, Eq. (5) shows that the transmitting coil provides not only the inductive electromotive force, −j*ωMI’*_1_, but also a reflected impedance, given by (*ωM*)^2^/*Z*_1_, for the receiving coil. Note that the actual current in the transmitting coil is *I*_1_ when the receiving coil is presented. Because the impedances *Z*_1_ and *Z*_2_ and the reflected impedances (*ωM*)^2^/*Z*_1_ and (*ωM*)^2^/Z_2_ may be complex and may contain a large imaginary component, impedance matching of this two-coil system can be challenging.

When the self-resonant frequencies formed by the two coils are the same, and the operating frequency is exactly equal to that self-resonant frequency, the impedances and the reflected impedances of the two closed circuits are all real numbers. The impedance matching condition required can be written simply as

6 $${R}_{S}={(\omega M)}^{2}/{R}_{L}$$

If the reflected impedance of the load resistance seen by the source is equal to the source resistance, then the two-coil system meets the impedance matching requirement and the transmission efficiency reaches a maximum. In Eq. (6), the reflected impedance is shown to be proportional to the second power of the mutual inductance. This means that the impedance matching condition is highly sensitive to the transmission distance.

**Figure 2:**
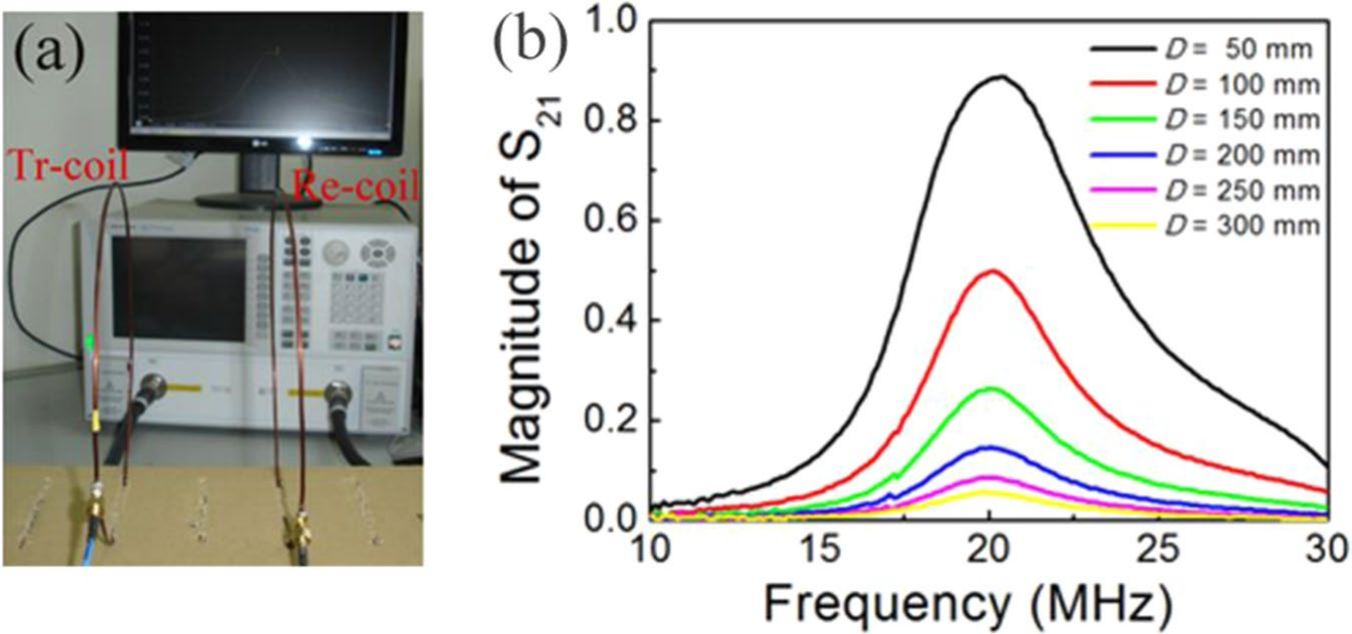
(**a**) Photograph of the two-coil system and the measurement equipment used. The left coil is the transmitting coil and the right coil is the receiving coil. The coupling distance D between the transmitting and receiving coils can be tuned. (**b**) Measured values of scattering parameter *S*_21_ at various coupling distances.

Figure 2(a) shows a photograph of the two-coil system and the measuring equipment used to test it. The copper coil is composed of a single turn. The radius of the copper coil, which has a cross-sectional radius of 1 mm, is 100 mm (the inductance is 590 nH, as calculated using the following formula^42^: *L* = µ_0_*a*[ln(8*a*/*d*)-2]), and a lumped capacitor with a value of 100 pF is connected to the coil to achieve resonance, thus meaning that the resonant frequency of the copper coil is 20.7 MHz. Because this two-coil system is actually a two-port network, the impedance matching can be presented in terms of the linear magnitude of the scattering parameter *S*_21_. A vector network analyzer (Agilent PNA E8361C) is used to measure the magnitude of *S*_21_. In Fig. 2(a), the input and output ports are connected to the transmitting and receiving coils, respectively. Both the source resistance and the load resistance of the system are 50 Ω.

In the experiment, we found a peak value of the magnitude of *S*_21_ at a frequency of 20.5 MHz. At this frequency point, the reflected impedance of the load becomes a real number and has a value close to that of the source resistance. When the peak reaches unity, perfect impedance matching of the system is achieved. This means that the system has reached a critical coupling state, and the critical coupling distance is less than 50 mm. When the coupling distance moves little by little away from the critical coupling position, the perfect impedance matching condition is then gradually destroyed. The magnitude of *S*_21_ versus coupling distances ranging from 50 mm to 300 mm is plotted in Fig. 2(b). The results are consistent with those of the theoretical analysis.

### Broadband impedance matching of three-coil system

**Figure 3:**
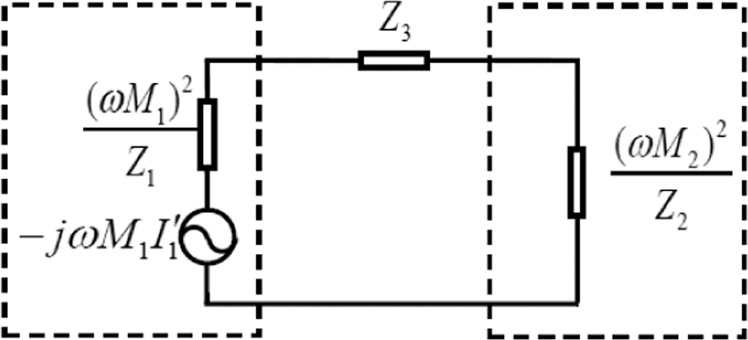
Equivalent circuit of the three coupled LC resonant coils at the resonant frequency.

From the results above, we know that for a fixed (in terms of operating frequency, coil size and load resistance) two-coil system, any coupling distance that exceeds the critical coupling distance will lead to impedance mismatch. To solve this troublesome problem, we propose a new impedance matching mechanism based on introduction of a third LC resonant coil into the mismatched two-coil system. Importantly, broadband impedance matching was observed experimentally when using this method. We first analyze this matching mechanism using an equivalent circuit model. Using the interrelation between the two coils as described in Eqs. (4) and (5), the equivalent circuit for the three-coil resonant system can be determined. Figure 3 shows this equivalent circuit at the resonant frequency. The dashed frames on the left and right represent the contributions of the transmitting and receiving coils to the third coil, respectively, while Z_3_ represents the impedance of the third coil. It should be noted here that direct coupling between the receiving and transmitting coils does exist, but it is very small because the electromagnetic field generated by a current carrying coil is an evanescent field and thus can be ignored in the three-coil system. The mutual inductances between the third coil and the transmitting and receiving coils are denoted by M_1_ and M_2_, respectively. At the resonant frequency, the third coil impedance Z_3_ is assumed to be zero, and the reflected impedances of the load resistance R_L_ and the source resistance R_S_ as seen by the third coil are (ωM_2_)^2^/R_L_ and (ωM_1_)^2^/R_S_, respectively. Therefore, by adjusting the positioning of the third coil, the impedance matching condition can be achieved, even if the coupling distance between the transmitting and receiving coils is much greater than the critical coupling distance.

**Figure 4:**
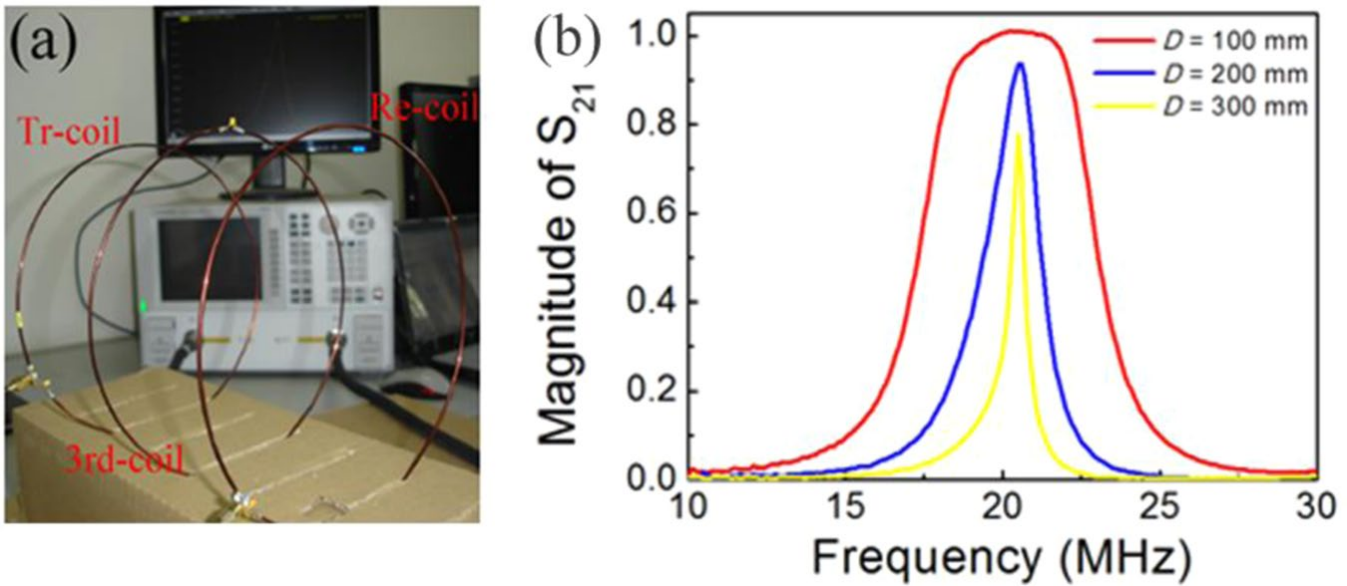
(**a**) Photograph of the three-coil system. The left coil is the transmitting coil; the right coil is the receiving coil; and the center coil is the inserted third coil. (**b**) Measured characteristics of scattering parameter *S*_21_ at different values of coupling distance *D*.

Figure 4(a) shows the manufactured three-coil resonant system. The coils are identical to those shown in Fig. 2(a). Both the power internal resistance *R*_S_ and the load resistance *R*_L_ have values of 50 Ω. The third coil is placed at the exact center position of the system (with *M*_1_ being equal to *M*_2_) and thus the system meets the impedance matching requirements in terms of the theoretical analysis described above. Figure 4(b)shows the measured results for the variation of the linear magnitude of scattering parameter *S*_21_ with different values of distance *D* between the transmitting and receiving coils. When compared with Fig. 2(b), the figure clearly shows that the peak values of the magnitude of *S*_21_ have improved significantly. For example, at a distance *D* = 100 mm, the frequency range over which the magnitude scattering parameter *S*_21_ is greater than 0.9 is 5 MHz, ranging from 18 MHz to 23 MHz. However, *S*_21_ barely reaches 0.5 at the resonant frequency of 20.5 MHz alone in the two-coil system. Therefore, broadband impedance matching of the proposed system composed of three coupled LC resonant coils has been achieved. Theoretically, for any transmission distance *D*, the peak value of the magnitude *S*_21_ can reach unity as long as *M*_1_ is equal to *M*_2_ and the dissipation of the third coil is negligible. This means that perfect impedance matching is realized when using the proposed three-coil resonant system, regardless of the magnitude of the transmission distance. In fact, as the distance *D* increases, peak value of the magnitude of *S*_21_ decreases, as shown in Fig. 4(b). This occurs because the reflected impedances of both the power internal resistance and the load resistance reach the order of magnitude of the resistance of the third coil. Under these circumstances, a considerable proportion of the power is then dissipated by the third coil. The nonzero impedance of the third coil at the resonant frequency should also be taken into consideration as part of the impedance matching issue. However, the practical application of metasurface-based broadband transmitters and absorbers with overly thick layers would be restricted. Impedance matching over a large coupling distance is therefore unnecessary and is not considered here. Thus far, the feasibility of broadband impedance matching has been proved both theoretically and experimentally. Although the feasibility of the method has been proved using an LC resonant coil, the method could be extended to any electromagnetic resonator, such as a metal pattern resonator or a dielectric cylinder resonator, which is the unit cell resonator that is commonly used in metasurfaces.

## Discussion

We have demonstrated broadband impedance matching through use of coupled LC resonant coils. By adding a third resonant coil at the center of the mismatched two-coil system, in which the distance between the transmitting and receiving coils is 100 mm, the peak magnitude of the scattering parameter *S*_21_ is significantly enhanced to unity. Furthermore, the frequency zone in which the parameter *S*_21_ is greater than 0.9 is expanded and ranges from 18 MHz to 23 MHz; this compares favorably with the two-coil system, in which the peak magnitude of *S*_21_ only reaches 0.5 at the resonant frequency of 20.5 MHz. The results reported here prove the feasibility of broadband impedance matching using these coupled resonators. Therefore, the matching scheme presented in this paper will provide a useful guide for realization of broadband perfect absorbers and transmitters.
